# Desensitization of the Cough Reflex Induced by Corticosteroids in Ovalbumin-Sensitized Rabbits During Artificial Limb Exercise

**DOI:** 10.3389/fphys.2020.00466

**Published:** 2020-05-13

**Authors:** Simon Valentin, Bruno Chenuel, Silvia Demoulin-Alexikova, Bruno Demoulin, Delphine Gérard, Laurent Foucaud, Mathias Poussel

**Affiliations:** ^1^EA 3450 DevAH – Development, Adaptation and Disadvantage, Cardiorespiratory Regulations and Motor Control, Université de Lorraine, Nancy, France; ^2^Department of Pneumology, CHRU Nancy, Nancy, France; ^3^Pulmonary Function Testing and Exercise Physiology, CHRU Nancy, Nancy, France; ^4^Laboratory of Hematology, CHRU Nancy, Nancy, France

**Keywords:** cough reflex, airway inflammation, asthma, exercise, corticosteroids

## Abstract

**Introduction:**

Cough is a major symptom frequently experienced during exercise, mainly in asthmatic patients. Inhaled glucocorticoids represent the keystone treatment in the management of asthma, but little is known about interactions between cough and exercise, especially in controlled patients. During exercise, cough reflex (CR) appears downregulated in healthy animal models whereas a lack of desensitization of CR has been shown in ovalbumin-sensitized animal models, mimicking asthmatic disease.

**Aims and Objectives:**

The goal of our study was to clarify the potential modulation of the CR induced by inhaled corticosteroids (CS) in ovalbumin (OVA) sensitized rabbits during artificial limb exercise.

**Materials and Methods:**

Seventeen OVA sensitized rabbits were studied. Among them, 9 were treated with CS delivered intravenously (OVA-Corticoids). The ventilatory response to direct tracheal stimulation, performed at rest and during exercise, was determined to assess the incidence and the sensitivity of the CR. Broncho-alveolar lavage (BAL) and cell counts were performed to determine the level of airway inflammation. Exercise was mimicked by electrically induced hindlimb muscular contractions (EMC).

**Results:**

Compared to rest values, EMC increased minute ventilation by 28% without any decrease in respiratory resistance (Rsr). Among 322 tracheal stimulations, 172 (53%) were performed at rest and 150 (47%) during exercise. The sensitivity of CR decreased during artificial limb exercise compared to baseline in OVA-Corticoids rabbits (*p* = 0.0313) while it remained unchanged in OVA rabbits (*p* = NS).

**Conclusion:**

Corticosteroids appear to restore the desensitization of the CR in OVA sensitized rabbits during artificial limb exercise, suggesting the potential role of airway inflammation in the pathophysiology of cough during exercise in asthmatics.

## Introduction

Chronic cough remains a real public health problem due to its high prevalence and lack of effective treatment, since its pathophysiological determinants are not clearly elucidated. Indeed, chronic cough is the cause of 6% of consultations in the general practice and up to 30% in pulmonology, growing with the aging of the population (Jankowski, 2006). During exercise, cough is a very frequent symptom for both athletes and non-athletes and can be associated with other respiratory symptoms (dyspnea, wheezing, or chest tightness) (Couillard et al., 2014). Cough is also frequently associated with diseases involving pulmonary eosinophilic inflammation [asthma (Wenzel, 2006), allergy or eosinophilic bronchitis]. Furthermore, cough can be the unique symptom of asthma, then called cough-variant asthma. In these patients, the bronchial wall is mainly infiltrated with eosinophilic cells (Adamko et al., 2003). Physical activity is a major stimulus for asthma symptoms for many patients and cough and bronchoconstriction typically worsens during or after exercise (Parsons et al., 2013). CS are the key treatment in type 2 T helper (Th2) asthma phenotype and more broadly in any respiratory disease that involves eosinophilic inflammation. Indeed, CS can reduce exacerbations and mortality due to Th2 asthma phenotype and improve symptoms, including cough in asthmatic adults (O’Byrne et al., 2001). In exercise-induced bronchoconstriction (EIB), CS are strongly recommended in addition to bronchodilators, resulting in the reduction of symptoms (Poussel and Chenuel, 2010; Parsons et al., 2013).

The pathophysiology of cough during exercise remains unclear but undoubtedly involves numerous mechanisms suggesting that cough exhibits plasticity at both peripheral and central levels (Bonham et al., 2004; Widdicombe and Singh, 2006; Aggarwal et al., 2018). Previous studies have proven that CR is decreased during exercise in healthy subjects (Lavorini et al., 2010; Demoulin-Alexikova et al., 2017) as well as in animal models (Poussel et al., 2014), even the precise underlying mechanisms are still debated. In contrast, CR sensitivity during exercise was unchanged (i.e., lack of downregulation) in ovalbumin (OVA)-sensitized rabbits (eosinophilic bronchial inflammation mimicking classical asthmatic inflammation) (Tiotiu et al., 2017), suggesting the contribution of bronchial eosinophilic inflammation in the modulation of CR in asthmatics during exercise.

The main goal of our study was to clarify the possible influence of CS on CR sensitivity during exercise in OVA-sensitized rabbits. Ventilatory responses to mechanical stimulation of trachea were identified from airflow and electromyography of abdominal muscles, enabling us to differentiate CR from ER. CR is a powerful airway defensive mechanism characterized by 3 respiratory successive phases (Inspiration-Compression-Expulsion) (Leith, 1985) and must be differentiated from ER which does not have an initial inspiratory phase (Lötvall, 1994). Since these 2 reflexes have different neuro-anatomical organization and physiological function, their distinction is essential in the current literature (Widdicombe and Fontana, 2006; Varechova et al., 2010, 2012). Finally, the use of validated and reproducible methodology of mechanical stimulation of trachea, elaborated in our laboratory permitted us to assess the sensitivity of defensive reflexes to mechanical stimulation by using several mechanical stimulation durations.

We hypothesized that CS in OVA-sensitized rabbits could restore the desensitization of CR during artificial limb exercise.

## Materials and Methods

All animal procedures (housing and experiments) were approved by the Lorraine University Committee for the Use and Care of Laboratory animals (November 23, 2017) and by the Ministry of Higher Education, Research and Innovation (March 01, 2018) under the reference “APAFIS#11197-2017090810597513v5.” Procedures were performed between March 2018 and November 2018. Seventeen anesthetized, tracheotomized and spontaneously breathing New Zealand adult rabbits (weight: 2.994 ± 0.20 kg) were studied (SARL HYCOLE. Route de Villers Plouich, 59159 MARCOING, France)^1^. Rabbits were randomized in 2 groups: “OVA-Corticoids IV” group (*n* = 9) and “OVA-Control” group (*n* = 8), based on their initial allocation cage. There was no difference between the two groups in terms of sex and weight.

### Ovalbumin Sensitization Protocol (Figure 1)

As previously described (Tiotiu et al., 2017), rabbits were sensitized the month before the acute experiment (performed on day 29 on the trial calendar) according to the following protocol:

**FIGURE 1:**
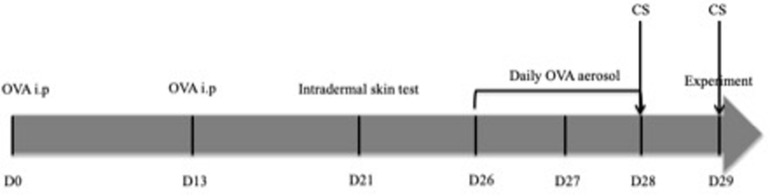
Timeline of pre-experimental interventions. CS only concerns rabbits in the “OVA-Corticoids IV” group. (i.p., intra-peritoneal injection; CS, corticosteroids; OVA, ovalbumin; D0, Day 0).

- Days 0 and 13: intra-peritoneal injections of a solution containing 1 mL of saline in which 0.1 mg of OVA and 10 mg of aluminium hydroxide Al(OH)3 were dissolved.
- Days 23– 25: animals were exposed to saline aerosols to get them used to the nebulizer system.
- Day 26–28: animals were exposed to OVA aerosols (2.5 mg/mL) for 20 min each nebulization.

Aerosols were administered in a closed box in Plexiglas^®^ with an ultrasonic nebulizer (SYST’AM^®^, LS290).

### Ovalbumin Sensitization Evaluation

#### Intradermal Skin Tests (Figure 1)

Ovalbumin sensitization was checked by intradermal skin tests 7 days before the experiment (i.e., day 21). Thus, 100 μL of an OVA solution (200 μg/mL) was injected subcutaneously into the skin of the animal’s dorsal region, previously cleaned and shaved. Intensity of the reaction was measured 24 h after the injection (i.e., day 22), by measuring the wheal area of the induration extent. Saline sham injection was performed at the same time, following the same protocol. Rabbits without a skin reaction at the OVA injection site at 24 h have been excluded from the study.

### Bronchoalveolar Lavage (BAL)

Bronchoalveolar lavage was performed after the tracheal stimulation protocol and following animal euthanasia (Tiotiu et al., 2017). Using a polyethylene-190 catheter placed through the endotracheal cannula, 5 ml of “HEPES” solution (140 mM NaCl, 5 mM KCl, 1 mM MgCl2, 10 mM glucose, and 10 mM HEPES dissolved in distilled water; pH = 7.4) was injected 3 times, followed each time by a gentle aspiration (total quantity of liquid injected of 15 ml). BAL liquid was collected in a tube placed in ice and filtered with a nylon cloth perforated with 60 μm holes to remove mucus. After carrying out a dilution adapted to the cell count of the BAL, the liquid was centrifuged (10 min, 600 rpm) (Cytospin AutoSmear OF-120E). The cytological material obtained was then stained by the May-Grünwald-Giemsa technique to allow cell count under optical microscope.

### Cell Count Under Optical Microscope

The slides obtained were studied with an Olympus microscope equipped with a x40 lens and recorded by an Olympus CDD camera in a 1360 × 1024 pixels format. On each blade, at least 100 cells were counted and differentiated according to their morphological characteristics between: macrophages, lymphocytes, neutrophils, eosinophils, basophils, and monocytes. All blades were read blindly by a confirmed cytologist.

### Corticosteroids Administration (Figure 1)

In the group “OVA-Corticoids IV,” animals received two intravenous injections, via the marginal vein of the ear, of methylprednisolone (Solumedrol^®^, ZOETIS) at a dose of 1mg/kg. The first injection was delivered 24 h before the acute experimentation (day 28) and the second 1 h before the final acute experiment (day 29).

### Anesthesia and Surgical Preparation

Anesthesia was performed using solutions of urethane (50 mg.mL^–1^; 12.5 g urethane in 250 mL of saline) and urethane-chloralose (50 mg.mL^–1^; 12.5 g urethane and 12.5 g chloralose in 250 mL of saline). Induction was obtained by intravenous injection (marginal vein of the ear), of 1 mL.kg^–1^ of urethane and 1 mL.kg^–1^ of urethane-chloralose. Depth of anesthesia was regularly checked (every 30 min) by ensuring the presence of a corneal reflex, but the absence of ear retraction and reaction to the ear pinching, evidences of optimal anesthesia. In case of insufficient anesthesia, a maintenance dose of 0.2 mL.kg^–1^ urethane and 0.2 mL.kg^–1^ urethane-chloralose was injected through the catheter in place. At the end of the experiment, euthanasia was obtained by a lethal dose of Doléthal^®^ (5 mL bolus, Vétoquinol SA, Lure, France).

A sagittal incision of the skin, followed by dissection of the subcutaneous and muscular tissues was performed, in order to respect the vagal nerves. The trachea was then transversely sectioned, and animals were tracheotomized and intubated with a steel tracheostomy cannula, adapted to the size of the trachea. This surgical preparation under anesthesia allowed the connection of tracheotomy cannula to a pneumotachograph (No. 0 Fleisch pneumotachograph with linear range ± 250 mL/s) and to the mechanical stimulation apparatus. The pneumotachograph was calibrated before each experiment using a 20 ml calibration syringe. The body temperature was continuously recorded with an electronic thermometer (Physiotemp Instruments, YSI 402 Clifton, NJ, United States) by intrarectal route to maintain the temperature at 38°C. Electrocardiogram electrodes were also placed on the chest to record heart rate (Varechova et al., 2010; Poussel et al., 2014; Tiotiu et al., 2017).

### Rhythmic Electrical Stimulation of Muscle Contraction (EMC)

On shaved posterior legs of anesthetized rabbits, surface electrodes were placed on the skin (Dura-Stick Premium, REF 42205, DJO, United States) in order to perform EMC. Electrodes were connected to an electrical stimulator (Neuro Trac Rehab, Verity Medical Ltd, United Kingdom). Muscle contractions were triggered by repetitions of stimulations of 2 s separated by free intervals of 2 s. The intensity oscillated between 10 and 30 mA with a growth and decrease time of 0.5 s. The stimulation was maintained for 3–4 min, in order to induce hyperventilation similar to that obtained during a moderate physical exercise (ventilation at least 20% higher than rest value).

### Electromyography

Electromyography of abdominal muscles was performed according to Basmajian and Stecko (1963) using bipolar electrodes (A-M Systems, INC, Carlsborg, WA, United States) inserted either through transverse muscles of the abdomen, or external oblique muscles to objectively confirm movements associated with the ventilatory response induced by tracheal stimulation.

### Tracheal Mechanical Stimulation

A Silastic^®^ semi-rigid catheter (0.7 mm, OD Metric) was inserted into the tracheotomy cannula, along the tracheal wall to trigger respiratory reflexes. The lower end of the catheter was placed 3 to 4 cm from the upper extremity of the cannula, facing the lower end of the trachea or the carina. The upper end of the catheter was connected to a rotating electrical motor (low voltage DC motors 719RE280, MFA/Comodrills, United Kingdom), that cause tracheal mechanical stimulation by scraping the tracheal wall. Duration of stimulations were: 50 ms (corresponding to a single rotation), 150, 300, 600, and 1000 ms.

### Signal Analysis

Acquisition of the analogic signal was carried out by a PowerLab^®^ 30 series system (ADInstruments, ML880 PowerLab 16/30) with an acquisition frequency of 200 Hz and a 16-bit sampling resolution. The data, once digitized, were stored on a disk and then later analyzed using the LabChart7-Pro software (ADInstruments, v 7.1). Ventilated flow ($\dot{V}$E) was measured at the tracheostomy cannula using a pneumotachograph (Metabo, Hepalinges, Switzerland), and integrated to obtain the volume. Tidal volume (V_T_) and flow were recorded continuously during the experiment. Rsr was measured by an adaptation of the oscillation technique (Delacourt et al., 2000; Varechova et al., 2010; Poussel et al., 2014; Tiotiu et al., 2017). More precisely, the airway opening was attached to a 3-way connector. A loudspeaker (ZR4009A, Bouyer, Montauban, France) connected to one end of the 3-way connector oscillated the transrespiratory pressure at a frequency of 20 Hz. The animal then breathed spontaneously through a high inertance tubing connected to the second connector, while a constant flow source flushed the breathing circuit with fresh air through the third connector to prevent CO_2_ accumulation. Rrs was computed from the real part of the complex airway pressure-flow ratio at 20 Hz.

Before tracheal stimulation, reference values (rest and exercise) were recorded by averaging the respiratory variables (V_T_, $\dot{V}$E_MAX_: peak expiratory flow) on 3 consecutives respiratory cycles. Responses to tracheal stimulation were assessed according to changes in V_T_ and $\dot{V}$E_MAX_ from the reference values. For each of these parameters, standard deviation (SD) was also calculated. A significant defensive response to the mechanical tracheal stimulation was considered when the ventilatory parameters were outside the 99^*th*^ percentile (above mean + 3 SD). Three different responses to stimulation were observed: CR (defined by a significant increase in V_T_ and $\dot{V}$E_MAX_), ER (defined by an isolated increase in $\dot{V}$E_MAX_, not preceded by an increase in V_T_) and absence of response (NR), characterized by the absence of modification of the V_T_ and $\dot{V}$E_MAX_.

The primary endpoint is the determination of the cough reflex threshold (CT), defined as the shortest stimulus duration able to trigger a CR response immediately after the tracheal stimulation. In case of multiple response (i.e., more than one expulsive event following the stimulation), only the first event is taken into account to determine the CT.

### Protocol (Figure 2)

A sequence consisted of data acquisition of both rest and artificial limb exercise (i.e., EMC) records. Following an initial sequence only aimed to assess Rsr, each rabbit underwent 3 sequences separated by 10 min of recovery. During each sequence, tracheal stimulations were performed at rest (3 to 4) and during artificial limb exercise (3 to 4). Tracheal stimulations were systematically separated by a period of 1 min, and delivered with different durations (50 ms, 150 ms, 300 ms, 600 ms or 1000 ms) during inspiration.

**FIGURE 2:**
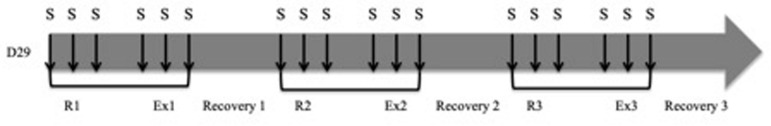
Chronology of experimental interventions: 3 consecutives sequences (R, rest; Ex, exercise; S, tracheal stimulation).

### Data Analysis

Statistical analysis was performed using JMP 13.0.0 software (2016 SAS Institute Inc). Respiratory variables (V_T_, $\dot{V}$E, and Rsr) were continuously recorded during rest and artificial limb exercise in order to compare the 2 groups. For comparisons, $\dot{V}$E and Rsr were averaged over a period of 60 s immediately preceding the beginning of exercise (rest $\dot{V}$E and rest Rsr) and also over the last 60 s of the exercise period (exercise $\dot{V}$E and exercise Rsr).

The incidence and responses to mechanical tracheal stimulation in both conditions (rest and exercise) were analyzed by a Chi 2 (χ2) or a Fisher test. Analysis of variance (ANOVA) was used for the comparison of respiratory variables. Comparison (rest versus exercise) of the values of CT was performed with the non-parametric Wilcoxon test. The significance threshold used was *p* < 0.05. Results are expressed in mean ± Standard Deviation.

## Results

### OVA Intradermal Skin Tests and BAL Fluid Count

All (*n* = 17) OVA sensitized rabbits showed a positive OVA intradermal injection challenge with a mean wheal area of 409.7 mm^2^ ± 395.3 mm^2^ (individual values are presented in Table 1). BAL fluid count showed no difference in eosinophil (3.1% vs 4.8%) and neutrophil (2.8% vs 3%) cells between the groups (respectively “OVA-Corticoids IV” group vs “OVA-Control” group; *p* = NS).

**TABLE 1:** Wheal area of the induration extent measured 24 h after the intradermal injection of 100 μL of an OVA solution (200 μg/mL) in OVA sensitized rabbits (expressed in mm^2^).

**“OVA-Corticoids IV” group *n* = 9**	**Wheal area (mm^2^)**	**“OVA-Control” group *n* = 8**	**Wheal area (mm^2^)**
**Rabbits**		**Rabbits**	

1	95	1	183
2	471	2	299
3	471	3	87
4	113	4	143
5	452	5	79
6	731	6	79
7	1590	7	95
8	660	8	638
9	779		

### Respiratory Resistance and Breathing Pattern at Rest and Exercise

Overall (*n* = 17), minute ventilation ($\dot{V}$E) increased from 916 mL.min^–1^ ± 186 mL.min^–1^ at rest to 1180 mL.min^–1^ ± 377 mL.min^–1^ during artificial limb exercise (+ 28%). Concomitantly, Rsr remained unchanged (Rest Rsr = 22.8 hPa.s.L^–1^ ± 5.3 hPa.s.L^–1^; Exercise Rsr = 22.7 hPa.s.L^–1^ ± 5.8 hPa.s.L^–1^). Rest and exercise minute ventilation were not statistically different (*p* = NS) between “OVA-Corticoids IV” group (respectively: Rest $\dot{V}$E = 903 mL.min^–1^ ± 238 mL.min^–1^; Exercise $\dot{V}$E = 1219 mL.min^–1^ ± 517 mL.min^–1^) and “OVA-Control” group (respectively: Rest $\dot{V}$E = 930 mL.min^–1^ ± 118 mL.min^–1^; Exercise $\dot{V}$E = 1136 mL.min^–1^ ± 123 mL.min^–1^). Rest and exercise Rsr remained unchanged (*p* = NS) between “OVA-Corticoids IV” group (respectively: Rest Rsr = 23.7 hPa.s.L^–1^ ± 5.9 hPa.s.L^–1^; Exercise Rsr = 21.9 hPa.s.L^–1^ ± 6.1 hPa.s.L^–1^) and “OVA-Control” group (respectively; Rest Rsr = 22.0 hPa.s.L^–1^ ± 5.2hPa.s.L^–1^; Exercise Rsr = 23.7 hPa.s.L^–1^ ± 5.9 hPa.s.L^–1^).

### Cough Threshold (Figure 3 and Table 2)

An overall of 51 sequences (including rest and artificial limb exercise) and 322 tracheal stimulations [172 (53%) at rest; 150 (47%) during exercise] were performed and analyzed in 17 rabbits [“OVA-Corticoids IV” group (*n* = 9) and “OVA-Control” group (*n* = 8)]. In “OVA-Corticoids IV” group, 174 tracheal stimulations were performed, respectively, 94 (54%) at rest and 80 (46%) during artificial limb exercise. In “OVA-Control” group, 148 stimulations were performed, respectively, 78 (53%) at rest and 70 (47%) during artificial limb exercise.

**FIGURE 3:**
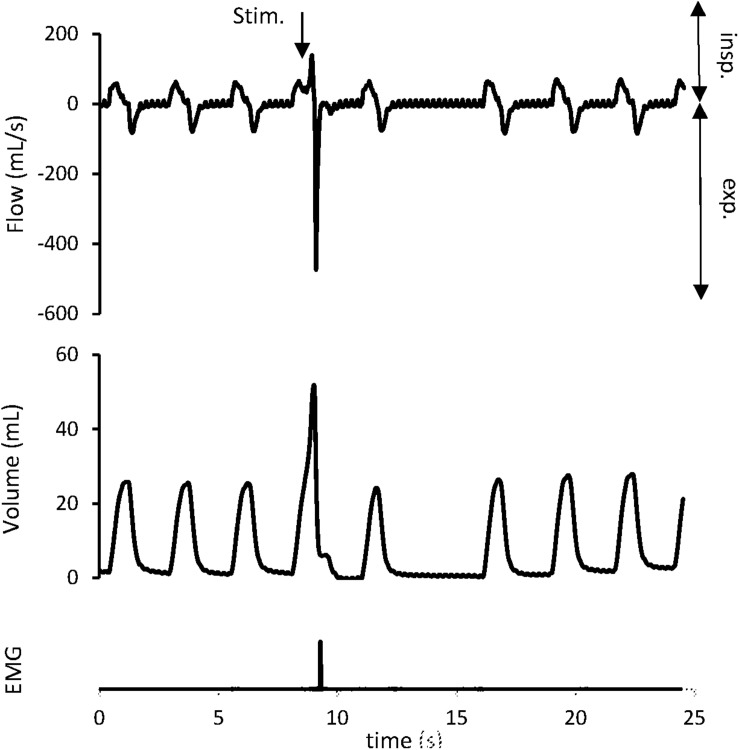
Cough reflex characterized by a concomitant increase in tidal volume (V_T_) and peak expiratory flow ($\dot{V}$E_MAX_). The downward arrow indicates tracheal stimulation (Stim.). Positive and negative airflow rates indicate inspiration (insp) and expiration (exp), respectively. Abdominal muscles electromyogram (EMG) also showed activity on the stimulation breath.

**TABLE 2:** Cough Threshold (CT) at rest and exercise in “OVA-Corticoids IV” group and “OVA-Control” group (expressed in ms).

**“OVA-Corticoids IV” group *n* = 9**	**CT (ms)**		**“OVA-Control” group *n* = 8**	**CT (ms)**	
**Rabbits**	**Rest**	**Exercise**	**Rabbits**	**Rest**	**Exercise**
1	150	300	1	50	50
2	300	300	2	300	300
3	300	600	3	150	50
4	150	150	4	300	300
5	50	50	5	300	300
6	50	150	6	300	300
7	50	150	7	50	150
8	50	150	8	50	50
9	50	50			

Cough reflex (Figure 3), ER, and NR were the 3 types of response encountered. Expulsive events were single (291/322; 90.4%), double (30/322; 9.3%), or triple (1/322; 0.3%). In the “OVA-Control” group, CT remained unchanged in 6 rabbits, increased in 1 rabbit and decreased in 1 rabbit supporting no modification of the CR sensitivity between rest and exercise (*p* = NS). In the “OVA-Corticoids IV” group, CT increased in 5 rabbits and remained unchanged in 4 rabbits indicating an increase of the CR sensitivity between rest and exercise (*p* = 0.0313). CT at rest and exercise in both groups are presented in Table 2.

## Discussion

Our study was designed to assess the possible influence of CS on the CR sensitivity during exercise in OVA-sensitized rabbits. The central finding of our work is that CS is able to restore the desensitization of the CR during artificial limb exercise in OVA-sensitized rabbits.

Cough has been extensively studied (Widdicombe et al., 1985; Widdicombe and Fontana, 2006) but little information is available regarding to its interactions with human activities, especially during exercise (Widdicombe et al., 2009). However, the emerging and now accepted concept of cough hypersensitivity syndrome (CHS) (Morice et al., 2014; Chung et al., 2016) clearly identify “exercise” as a cough trigger highlighting the significant role of “exercise” in cough (Gibson, 2019). Basically, the model of the CHS requires two key components that are a cough trigger able to activate the afferent limb of the CR (exposures or diseases such as, respectively, exercise or asthma), and cough hypersensitivity (such as laryngeal dysfunction or central sensitization). Overall, exercise and its concomitant adaptive physiological responses appears to modulate the CR (Widdicombe et al., 2009; Tiotiu et al., 2017). Clinical studies have already shown a down regulation (i.e., increase of CT) of the CR during exercise among both healthy subjects (Lavorini et al., 2010) and animal models (Poussel et al., 2014). In both studies, peripheral and central mechanisms of cough modulation are discussed, especially dealing with the consequences of the augmented sympathetic activity and the augmented breathing pattern (leading to increasing activity of slowly adapting pulmonary stretch receptors – SARs) during exercise but also with the contribution of a cough modulation at a central level. More precisely, the marked hyper-ventilation occurring during exercise leads to a significant heat and water loss responsible for changes in airway fluid osmolarity (Banner et al., 1984; Anderson and Smith, 1988). This induced hyper-osmolarity further leads to the release of various inflammatory mediators and to mucus hypersecretion able to stimulate both mechanically and chemically the nerve endings involved in the afferent cough pathway (Hallstrand et al., 2005; Gu et al., 2008). The hyperventilation state and its augmented breathing patterns (i.e., V_T_ and respiratory frequency) is also able to increase inputs from mechanoreceptors, leading to a modulation of the CR (Javorka et al., 1994). Finally, cough and breathing centers probably share common neural circuits at a brainstem level favoring the more appropriate response regarding to the prevailing needs (i.e., breathing during exercise and coughing at rest). Thus, during exercise, evidences seem to support that CR is down-regulated.

Cough is also frequently reported by asthmatic patients during exercise and also by some athletes developing EIB (Poussel and Chenuel, 2010; Turmel et al., 2012; Boulet and O’Byrne, 2015; Boulet et al., 2017; Boulet and Turmel, 2019) suggesting a consistent role of the underlying airway inflammation (Hazucha et al., 1996). In contrast to healthy subjects (and healthy animals), some studies showed a lack of down regulation of CR during exercise in asthmatic patients and animal models of airway inflammation. In a study conducted in asthmatic children, CR sensitivity (capsaicin cough challenge) was assessed before and after a standardized bout of exercise (Ferenc et al., 2018). Authors showed that CR sensitivity was not significantly changed after exercise in this asthmatic population supporting a lack of evident down regulation of exercise on cough. Interestingly, a study also showed a trend to a lack of down-regulation of cough (capsaicin inhalation challenge) after exercise in children (versus adults) questioning the role of atopy in the pediatric population (Demoulin-Alexikova et al., 2017). Atopy has also been shown to be related to an increase of the CR sensitivity in patients with allergic rhinitis (Pecova et al., 2005) or atopic dermatitis (Pecova et al., 2003) highlighting the important role of atopy in the modulation of cough. In a previous study, our team also showed a lack of desensitization of CR (tracheal mechanical stimulation) during exercise in OVA-sensitized rabbits (Tiotiu et al., 2017). Altogether, airway inflammation appears to modulate CR during exercise, even if the precise mechanisms still need to be elucidated. Eosinophilic inflammation for instance seams to play a particular role (Brozmanová et al., 2006). Interactions between eosinophils and the sensory nerves located in the airways appear to enhance cough via different mechanisms such as neuro-inflammation (Gu et al., 2008) or activation of the transient receptor potential cation channel subfamily V member 1 (TRPV1) (Chung et al., 2013). Other studies dealing with elite athletes also showed a high prevalence of cough during either prolonged exercise or when performed under specific exposures such as allergens, pollutants, nitrous oxides, or chlorine derivatives (Boulet et al., 2017; Boulet and Turmel, 2019). Among the underlying hypothesis, inhalation of high concentrations of the above substances have been shown to decrease lung function, induce symptoms (cough but also breathlessness and chest pain [Boulet and Turmel, 2019] and increase airway neutrophilic inflammation due to the mild but repetitive bouts of bronchial epithelial lesions induced by exercise (Yoshihara et al., 1995). The central finding of our present study is that CR is desensitized during artificial limb exercise in OVA-sensitized rabbits that received CS but not in OVA-sensitized rabbits that did not received CS (*p* = 0.0313). Our results therefore support the possible role of CS in the modulation of cough during exercise, restoring the downregulation of cough during exercise as previously showed in non-sensitized patients (Lavorini et al., 2010) or animal models (Poussel et al., 2014). Even if we didn’t show a lower inflammatory cell count in the BAL fluid in the “OVA-Corticoids IV” group compared to the “OVA-Control” group (see section “Results”), repeated intra venous methylprednisolone injection was performed in the “OVA-Corticoids IV” group. We could then speculate that either the posology (1 mg/kg) of CS and/or the duration of the treatment (i.e., 2 times during a 24-h period of time) were insufficient to lead to a significant diminution of the BAL fluid cell count. However, during our experiment, we didn’t asses any anti-inflammatory/inflammatory mediators that could have been more or less released with our CS injections, and that could have modulated the CR. Indeed, a study performed in competitive cross-country skiers (Sue-Chu et al., 2000) aimed to investigate effects of inhaled budesonide showed no significant change in cellular inflammation in the bronchial mucosa (i.e., in accordance with our results) but a decrease in IL-2 receptors and activated T-helper lymphocytes suggesting a possible modulation of CR via other mechanisms consecutive to CS administration. Another possible mechanism that could explain our results and that should also be considered are the effects of CS on neural activity (Nagao et al., 1986). Indeed, it has been shown that CS have direct effects (depending on the dose delivered) on neuronal networks (Wittstock et al., 2015). Even if it has not been specifically studied neither regarding to airways afferents nor to the respiratory network, CS may then directly influence the threshold of CR. Our study was not designed to explore more precisely the underlying pathophysiological mechanisms but it appears that CS is able to restore the downregulation of cough during exercise, certainly through peripheral, and more central mechanisms (Widdicombe et al., 2011). Considering our results under clinical aspect, we could support the possible interest of CS in the management of cough during exercise (Boulet et al., 2017). Few studies are, however, available regarding the effect of treatments on cough in the athlete mainly concluding that drugs had only poor or even no effects on exercise-induced respiratory symptoms (including cough) (Sue-Chu et al., 2000; Bordeleau et al., 2014; Boulet et al., 2017).

Bronchomotricity is also another factor putted forward in the modulation of cough during exercise. EIB has been associated to desensitization of airway cough receptors (Smith et al., 1991) therefore contributing, at least partially, to the down regulation of CR during exercise (Marchal et al., 2012; Poussel et al., 2014). On the other hand, airway smooth muscle contraction appeared ineffective to directly trigger cough (El-Hashim and Amine, 2005) but may indirectly enhance cough induced by mechanical stimulation of the respiratory tract (Widdicombe, 2001). In contrast to previous studies (Poussel et al., 2014; Tiotiu et al., 2017), we did not show any bronchodilation during artificial limb exercise with no change of the Rsr despite a 28% increase of minute ventilation. This lack of bronchodilation during artificial limb exercise in sensitized rabbits could then be considered as a surrogate of bronchoconstriction possibly contributing to the modulation of CR, shifting cough to an increased threshold in our “OVA-Corticoids IV” group. However, no statistical difference for Rsr at rest and exercise were found between both groups, whereas the CT (rest versus exercise) was changed between the 2 groups. This is in accordance with other studies (Lavorini et al., 2010) demonstrating that cough was increased during exercise without any bronchodilation supporting that additional mechanisms, apart from airway dilation/constriction are involved.

Some limitations should also be discussed and comparisons with other studies should be carefully considered. Indeed, in the field of cough, whether it’s for human or animal, the methodology often differs from a study to another. Cough can be triggered chemically or mechanically involving different sensors and afferent pathways. Considerable methodological variability exists in the manner to induce cough (Wallace et al., 2019) and in the bouts of exercise delivered (modality, duration, and intensity). Characteristics of the rotating electrical motor used in our study insured a rectangular pattern to the mechanical stimulus with immediate cessation of catheter rotation at off-timing. Our animal model therefore allows a discriminant mechanical stimulation during the breathing cycle and we previously show that stimulations occurring during inspiration favor CR (versus stimulation during expiration favoring ER) (Varechova et al., 2010), suggesting the importance of the balance of discharge of SARs and rapidly adapting receptors (RARs) during the breathing cycle on the nature of the expulsive event. Thus, our model appears to be fitted to more particularly study the CR. Our exercise protocol is also original, via rhythmic EMC of the posterior limb muscles (Poussel et al., 2014). Even if it did not allow all the integrated and coordinated responses occurring during real exercise, it however, mimics dynamic exercise. Moreover, activation of limb afferents can also influence cough, as electrical stimulation has shown to directly activated limb nociceptive afferents that could modulate the “cough network” (Duranti et al., 1991). However, our exercise protocol used a low intensity current (ranged from 10 to 30 mA) that has been shown to limit direct stimulation of afferent fibers and prevent nociceptive stimuli (Cross et al., 1982). Overall, this exercise protocol could be regarded as a valuable model to explore the possible interactions between cough and exercise. Another limitation of our study lies in the absence of a non-sensitized group of rabbits (i.e., saline treated without OVA sensitization) that should allow to further discuss the possible involvement of airway allergic inflammation in the modulation of CR during artificial limb exercise. Indeed, as our study supports a clear OVA sensitization of the rabbits regarding to the results of intradermal skin tests (Table 1), inflammation could be considered as moderate (even weak) regarding to the BAL fluid count results. The lack of OVA sensitization (i.e., negative OVA intradermal injection challenge and lower BAL inflammatory cell count) in a non-sensitized group would have allowed to compare the CR threshold during limb muscle contraction and further highlight the potential role of inflammation in the modulation of CR. Based on the hypothesis of an absence of inflammation coupled with a desensitization of the CR during limb muscle contraction of such a non-sensitized control group, it should have reinforced our results. However, this limitation could find at least a partial answer, based on the results of a previous study of our teams (Poussel et al., 2014) including a non-sensitized group that showed a desensitization of the CR. Altogether, these methodological elements and limitations should always be kept in consideration and suggest that caution is warranted before any extrapolation.

## Conclusion

In conclusion, our study shows that intravenous administration of CS appears to restore the desensitization of the CR in OVA-sensitized rabbits during artificial limb exercise. It therefore suggests the potential contribution of airway inflammation in the pathophysiology of cough during exercise in asthmatics especially in eosinophilic inflammatory phenotypes. Further studies are needed to more precisely explore the underlying mechanisms involved in the modulation of cough during exercise in inflammatory diseases.

## Data Availability Statement

The datasets generated for this study are available on request to the corresponding author.

## Ethics Statement

The animal study was reviewed and approved by the Lorraine University Committee for the Use and Care of Laboratory animals.

## Author Contributions

SV, BC, and MP contributed to the design and the formal analysis. SV, BC, BD, and MP contributed to the data curation. SV, BC, SD-A, BD, DG, LF, and MP contributed to the investigation, the methodology, and the writing of the original draft. SV, BC, SD-A, and MP contributed to the supervision. SV, BC, SD-A, BD, and MP contributed to the validation. SV and MP contributed to the writing, the reviewing, and the editing. All authors contributed to manuscript revision, read and approved the submitted version.

## Conflict of Interest

The authors declare that the research was conducted in the absence of any commercial or financial relationships that could be construed as a potential conflict of interest.

## Footnotes

1 http://www.hycole.com/

## Abbreviations

BAL: Broncho-alveolar Lavage
CHS: cough hypersensitivity syndrome
CR: cough reflex
CS: corticosteroids
CT: cough reflex threshold
EIB: exercise-induced bronchoconstriction
EMC: electrical stimulation of muscle contraction
ER: expiratory reflex
NR: no response
OVA: ovalbumin
Rsr: respiratory resistance
SD: standard deviation
$\dot{V}$E: ventilated flow
$\dot{V}$ E_MA__X_: peak expiratory flow
V_T_: tidal volume.
